# Structural Analyses of Polysaccharides Extracted from Cyanobacterial Extracellular Gels and Oriented Liquid Crystalline Microfiber Processing by Poly(vinyl alcohol)-Assisted Electrospinning

**DOI:** 10.3390/gels10050321

**Published:** 2024-05-07

**Authors:** Chizu Mitani, Maiko Okajima, Tomomi Ohashira, Mohammad Asif Ali, Toshiaki Taniike, Tatsuo Kaneko

**Affiliations:** 1 Graduate School of Advanced Science and Technology, JAIST, Nomi 923-1292, Japan; s2220029@jaist.ac.jp (C.M.); ohashira5112@gmail.com (T.O.); asifali@jiangnan.edu.cn (M.A.A.); taniike@jaist.ac.jp (T.T.); 2 Key Laboratory of Synthetic and Biological Colloids, School of Chemical and Material Engineering, Jiangnan University, Wuxi 214122, China

**Keywords:** polysaccharides, physical gels, extracellular matrix, cyanobacterium, liquid crystals, glucan, electrospinning, water-soluble polymers, stimuli response, sacran

## Abstract

Sacran is a supergiant cyanobacterial polysaccharide that forms mesogenic supercoil rods that exhibit liquid crystalline (LC) gels at deficient concentrations of around 0.5 wt%, and has several bioactive stimuli-responsive functions. Here, we attempted to form oriented microfibers of sacran by electrospinning, following structural analyses of the sacran rods. A heterogeneous acid-hydrolysis method using a protonated cation-exchange resin was adopted to examine the short-time exposition of concentrated acid to sacran rods. From the supernatant, the oligomeric fraction that was soluble in water and methanol was isolated. The oligomeric fraction had a main sugar ratio of *α*-Glc:*β*-Glc:*α*-Xyl:*β*-Xyl:*α*-Rha of 2:5:1.5:1.5:4 (Glc:Xyl:Rha = 7 (=4 + 3):3:4), and it was speculated that the sacran structure includes rhamnoglucan and xyloglucan (4:3), which are generally rigid enough to exhibit LC. To make oriented microfibers of LC sacran, solubility testing was performed on sacran to find good new solvents of polyhydroxy alcohols such as ethylene glycol, 1,2-propanediol, and glycerol. The oriented film was prepared from a sacran aqueous solution where calcium compound particles deposited on the film are different from polyhydroxy alcohol solutions. Although sacran could not form microfibers by itself, polymer composite microfibers of sacran with poly(vinyl alcohol) were prepared by electrospinning. Cross-polarizing microscopy revealed the molecular orientation of the microfibers.

## 1. Introduction

**Figure 1:**
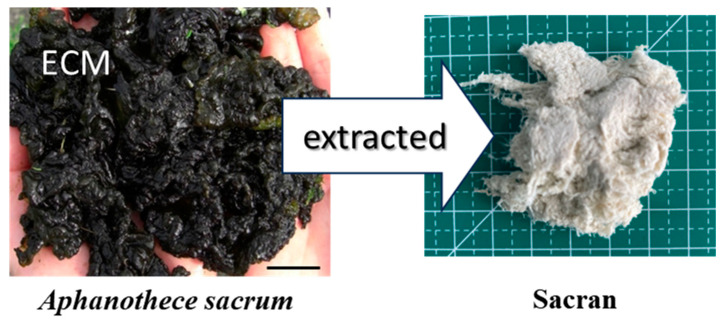
Appearances of a cyanobacterium colony (left photo) *Aphanothece sacrum* (scale bar 1 cm) and sacran cotton (right photo) extracted under alkaline condition from extracellular matrix (ECM) of the cyanobacterium (background scale 1 cm).

Water-soluble polysaccharides have been conventionally obtained from seaweed with a large amount of gel matrices [1,2,3,4,5]. Gelatinous biomaterials of a freshwater unicellular cyanobacterium, *Aphanothece sacrum*, are traditionally mass-produced in a local river of Japan [6,7,8,9,10,11,12], which has an abundance of a jelly extracellular matrix (ECM) which is a kind of gel with a high water content (97.5%) [13]. The ECM has good viscoelastic properties to reduce the damage of cell bodies from mechanical shocks in a river flow and to keep the algal body size above the centimeter range. We hypothesized that this ECM must mainly contain fibrous materials such as polysaccharides to reinforce itself. Therefore, we extracted polysaccharides, specifically, sacran, as a cotton-like foam from ECM of *A. sacrum* (Figure 1) and discovered that they consisted of sugar chains with a molecular weight (*MW*: 16 Mg/mol) [13] much higher than those of other polysaccharides used as superabsorbers [14,15]. The yield of sacran extraction ranged from 70 to 80 wt% in dried *A. sacrum* biomaterials, suggesting sacran as the main component of the ECMs. Most plants contain giant natural polymers such as polysaccharides, lignin, cellulose, hemicellulose, etc., and self-orientation phenomena are observed in plant cell walls, which may play a role in reinforcing plant bodies [16,17]. The extraction of intact celluloses with high *MW* is challenging due to its high crystallinity and insolubility. Moreover, the extraction can reduce *MW* to the scale of 0.1 Mg/mol, as evidenced by the *MW* values of the modified celluloses [18]. Although DNAs should have *MW* levels that are much higher than 10 Mg/mol, the *MW* is remarkably reduced to a scale of 1 Mg/mol during extraction processes [19]. On the other hand, *Aphanothece sacrum* exopolysaccharide was extracted by a mild condition based on alkaline dissolution and purification to keep high levels of *MW*, which induces a unique structural character of sacran. Transmission electron and atomic force microscopic studies of sacran extended by a spin-coating method on the glass revealed that their chains were very long, reaching lengths of 8 µm. In addition, the sacran formed a super-helical self-assembled microfiber and was used for a humidity-sensitive actuator [20]. The microfiber had a diameter of around 1 µm and consisted of a torsional self-assembled structure of nanofibers with diameters of around 50 nm. When one side of the microfibers was fixed, they drastically transformed into 2D structures like snakes and 3D twisted coils [20]. Such supercoils are rigid enough to exhibit a nematic liquid crystalline (LC) phase in water in concentrations ranging over 0.5 wt% [21]. The concentration is much lower than those of xanthan gum and shizophyllan, both of which are well-known high-performance LC polysaccharides, showing an LC phase above 6 wt% and 13 wt% [22], respectively. Even crystallite rods of charged celluloses with an average length of 115 nm show a critical LC concentration of 5 wt% [23,24]. The estimated aspect ratio of sacran mesogenic rods from Flory’s lattice theory [25] is around 1600, which is an anomalously high value compared to other LC polysaccharides such as xanthan gum and shizophyllan, which are 517 and 95, respectively [26]. The sacran chains with the LC phase efficiently adsorbed metal ions to form oriented hydrogels. This efficacy is attributed to sacran’s strong anionic properties, where 33 mol% anionic substitutes to sugar residues mean the accumulation of 30,000 negative charges per sacran chain. Trivalent metal ion adsorption to sacran caused the formation of shells on the gel beads due to the close packing of the sacran chains in their LC state (i.e., a sol core and a gel shell) [21]. Overall, sacran chains are very rigid megamolecules whose supercoils can be a strong mesogen activating in living *A. sacrum* ECMs showing LC-like opaque color. Then, the investigation of orientation and LC properties of sacran microfibers is important in the fields of material sciences and cyanobacterial biomechanics. Moreover, sacran has a unique glycosaminoglycanoid structure, considering functional groups such as sulfate, carboxylate, and N-acetylamide, leading to the inhibition of IgE and eosinophilic infiltration in mice, demonstrating sacran’s potential in the treatment of allergic dermatitis and anti-inflammatory pathways [27,28,29]. The beneficial health effects of sacran on skin eczematous disorders have been reported in human test subjects. Implementing a comparative clinical trial with a larger sample size was envisaged to confirm sacran’s efficiency and safety [30,31,32]. In spite of such attractive functions, sacran structure is yet insufficiently understood to optimize the fiber spinning condition. Moreover, microfibers as materials have never been prepared.

Here, we attempt to prepare oriented microfibers of the abovementioned stimuli-responsive polysaccharide, sacran, via electrospinning method, following sacran structural analyses. The sacran composite microfiber preparation can lead to not only the materialization of cyanobacterial polysaccharides in the field of LC devices and biomedical materials but also plant structural biologics and photosynthetic sciences since species of cyanobacteria have been regarded as excellent plant models [33].

## 2. Results and Discussion

### 2.1. Structural Analyses

Sacran has been extracted via the alkaline elution method from ECM gels of *Aphanothece sacrum* algal bodies (Figure 1). Extracellular carbohydrates occurring on the biological cell surfaces contain uronic acids. However, the sequence determination of uronate-abundant polysaccharides is complex due to the unusual acid instability of glycosidic bonds [34,35,36,37]. Structural analyses of sacran were previously attempted [38], but the fractionation was not successful in obtaining the proper oligomer specimens for the uronic acid instability for the following reasons: (1) too-high viscosity, hampering homogeneous hydrolyses, and (2) insolubility in strong acid solution. Although sacran was treated with 4M trifluoroacetic acid at 110 °C for 78 h, insoluble fractions remained while degraded black sugars appeared. The partial sugar component of successfully extracted fraction for sacran was Glc, Gal, Man, Rha, Xyl, and Fuc with a composition of 25.9:11.0:10.0:10.2:16.2:6.9, revealed from the analysis of gas chromatography–mass spectrometry (GC-MS) and GC. Differently from cellulose, sacran is a heteropolysaccharide composed of various sugar residues; therefore, its fractions of oligomers gave complex information about monosaccharide compositions as indicated here. However, sacran was non-crystalline and sufficiently soluble in water to use as a water absorbent.

**Figure 2:**
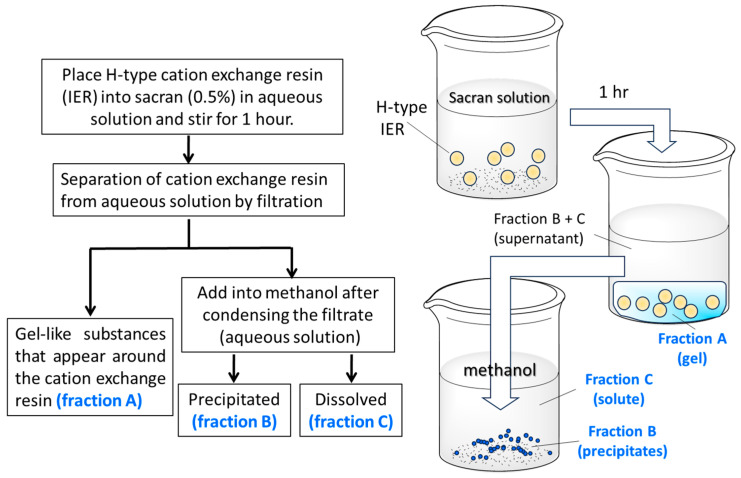
Process of fractionation by heterogeneous acid hydrolysis of sacran using H-type cation exchange resin (IER). Flow shown by text on the left and by illustration on the right.

**Figure 3:**
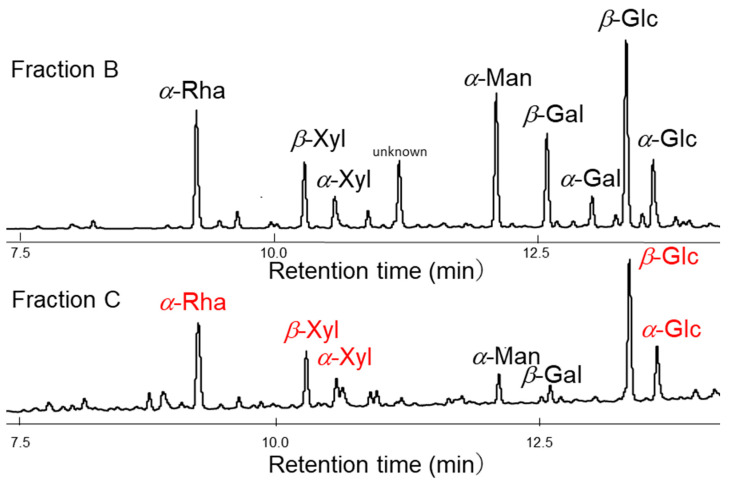
GC-MS chromatograms of sacran hydrolysates, Fraction B, and Fraction C. Red-marked monosaccharides in chromatogram of Fraction C are the main components. Glc: 1-O-methyl-tetra-O-trimethylsilylated glucose, Man: 1-O-methyl-tetra-O-trimethylsilylated mannose, Gal: 1-O-methyl-tetra-O-trimethylsilylated galactose, Xyl: 1-O-methyl-tri-O-trimethylsilylated xylose, Rha: 1-O-methyl-tri-O-trimethylsilylated rhamnose.

**Figure 4:**
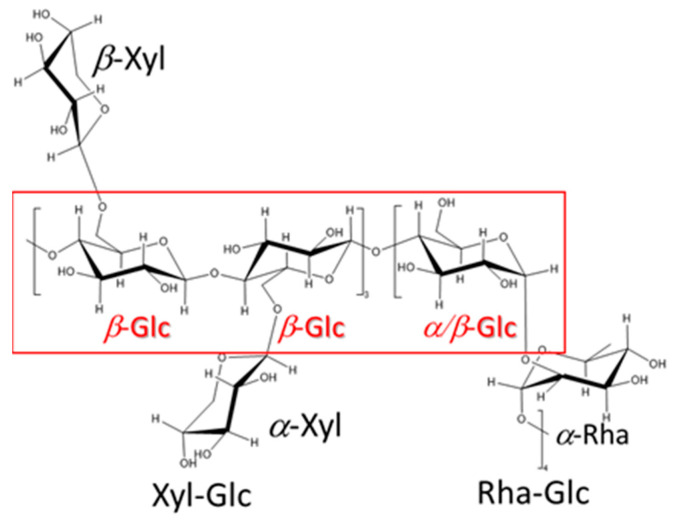
Proposed oligomer structures of sacran hydrolysate Fraction ·C· soluble in methanol after heterogeneous acid hydrolysis where *β*-1,4-glucan backbone was highlighted in red. The structure is putative to need additional confirmation.

Although homogeneous acid hydrolysis under various conditions failed in the unidentified repeating unit of sugar chains, a heterogeneous acid hydrolysis method has been proposed (Figure 2). The counter ions of the sulfonate group of a cation exchange resin were converted to H^+^ by hydrochloric acid treatment, and the resin was added to the aqueous solution of sacran. As a result, the sulfate and carboxyl groups of sacran were protonated and became a gel while incorporating the cation exchange resin beads (Fraction A). Next, the supernatant portion other than the gel was collected and concentrated, resulting in a highly viscous aqueous solution, and after methanol was added to this solution, the precipitates appeared. The precipitates were designated as Fraction B, and the fraction dissolved in methanol was designated as Fraction C. As for the insoluble Fractions A and B, an attempt was made to solubilize them with additional acid treatment. Complete solubilization of Fraction A was not possible even with additional acid treatment, and further analysis has not been conducted. Fraction B was completely solubilized by additional acid treatment, and was analyzed by GC-MS after methanolysis and trimethylsilylation. Fraction C was also analyzed by the analogous procedure with Fraction B. GC-MS chromatograms of Fractions B and C are shown in Figure 3. The monosaccharide composition of Fraction B was Glc, Man, Gal, Xyl, Fuc, Rha, GlcA, GalA, GalN, and unknown with a composition of 26.9:4.8:8.2:10.3:4.7:10.0:1.2:4.4:1.4:11.4, which was too complicated and considered to be a mixture of several oligomer components; further discussion was difficult to make. As for Fraction C, the sugar residue composition was 35.1:3.0:4.9:14.9:19.9 for Glc, Gal, Man, Xyl, and Rha. Since this is an almost integer ratio, it may include a repeating unit; therefore, the numbers below the decimal point were rounded off and divided by 5 to obtain the composition of 7:0.6:1:3:4 for Glc, Gal, Man, Xyl, Rha, respectively. Considering the small composition of galactose and mannose separately, the composition of Glc, Xyl, Rha was 7 (=3 + 4):3:4, respectively. This analysis suggested the existence of xyloglucan and rhamnoglucan as constituent oligosaccharides of sacran, as proposed in Figure 4. Further GC chromatogram analyses for *α*,*β*-isomer composition for sugar residues were carried out using isomer standard samples. The results are summarized as *β*-Glc:*α*-Glc = 5:2, *β*-Xyl, *α*-Xyl = 1:1, and *β*-Rha, *α*-Rha = 0:1. Then, the resulting ratio can be regarded as *β*-Glc:*α*-Glc:*β*-Xyl:*α*-Xyl:*α*-Rha = 5:2:1.5:1.5:4. Xyloglucan is a major constituent polysaccharide of hemicellulose [39], which is generally present universally in the primary cell walls of higher plants, and the main chain is composed of a very rigid *β*-1,4 glucan with *α*-1,6-linked xylose side chains. The sacran rigidity can be derived from the xyloglucan structure as shown in Figure 4. It is considered that sacran has similar structures to rhamnoglucan produced by Spirulina [40], which is also a cyanobacterium that produces sulfated polysaccharides. The rhamnoglucan structure is shown on the right side of Figure 4. Since galactose and mannose exist in small compositions of 0.6 and 1.0, it is speculated that galactose and mannose exist randomly as side chains. This suggests that the sacran chains may mainly contain a triad of rigid 1,4-*β*-glucan. The structure proposed in Figure 4 has no contradiction with the isomer ratio of monosaccharides. Although this structure is putative to need additional confirmation such as NMR, it can explain why sacran exhibits hydrogen bond interactions between sugar chains like hemicellulose, and also the presence of hydrophilic side chains makes it water-soluble, unlike hemicellulose. In other words, it can be discussed that sacran has a structure that makes it easy to become a fiber. As stated in the previous paper, sacran has anions of about 33%, such as sulfate groups, and then sacran can be an ionic cellulose analog [21]. The results can motivate us to obtain sacran microfibers by electrospinning.

### 2.2. Electrospun Microfibers

**Figure 5:**
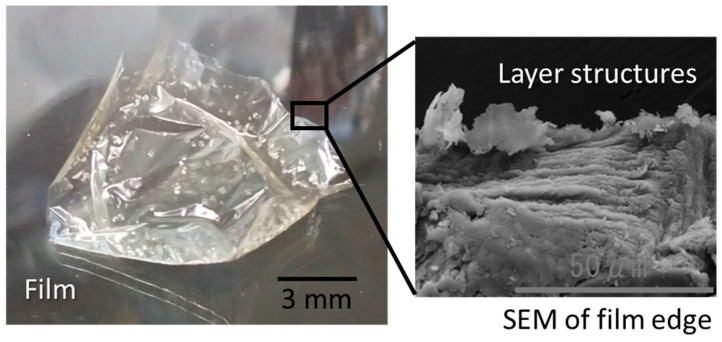
Film formed by electrospinning apparatus over sacran aqueous solution. Right picture: SEM of film edge showing striped texture of layer structures.

A solubility test was conducted to select the solvent used for electro-spinning. As seen in Table 1, it was found that sacran was soluble in polyhydroxy alcohols in addition to water and DMSO. Accordingly, we dissolved sacran in water, ethylene glycol, and 1,2-propanediol, which have relatively low boiling points, and tried electrospinning. However, the solvents did not completely dry away during flying, and the solution remained on the targeting glass substrate. When the solutions were dried, a film was formed as shown in Figure 5, where an SEM image of the film edge implied in-plane orientation. Previously, we reported the formation of sacran films oriented in-plain via slow-drying aqueous solution owing to LC polysaccharides [38]. Here, the formation of oriented sacran films under different conditions is noteworthy. From SEM, one can see that the band texture including layer structures with a distance ranging from 1 to 2-μm is much shorter than the sacran length, which is larger than 25 μm, as derived from a unit number (ca. 10^5^ number of monosaccharides) and a monosaccharide size (0.25 nm). Then, the sacran chains lay down cooperatively along the substrate.

### 2.3. Electrospun Microfibers

**Figure 6:**
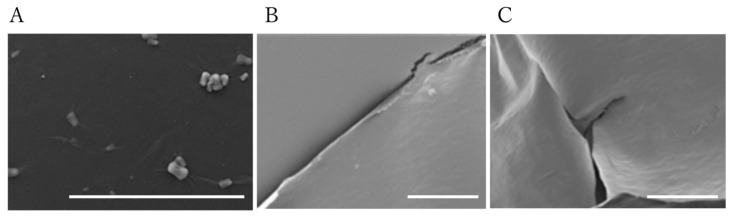
SEM images of sacran film surfaces formed in electrospinning machine. (**A**) Aqueous solution, (**B**) ethylene glycol, (**C**) 1,2-propanediol. Scale bars: 100 μm.

**Figure 7:**
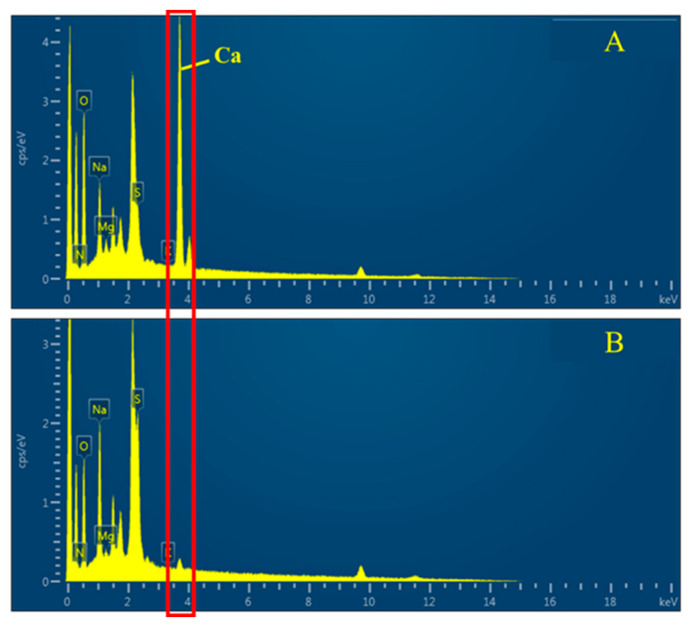
EDX spectra of particles formed on sacran film surface prepared over aqueous solution. (**A**) Particle; (**B**) film area without particles. Red square: Ca element.

SEM and EDX surface analyses of the sacran film were carried out. SEM images (Figure 6) reveal that particles appearing on the sacran film surface formed over an aqueous solution, while smooth surfaces of the films were prepared over ethylene glycol and 1,2-propanediol solutions. EDX analysis of particles (Figure 7) revealed that the particles were mainly composed of calcium, as it can be seen that only a calcium peak appeared compared to the film without particles. When the parent cyanobacterium cells, *Aphanothece sacrum*, are growing, they absorb a large quantity of calcium ions. Sacran chains include calcium ions as counter ions of carboxylate and sulfate groups.

Although the presence of calcium ions is negligible for any other evaluation and functionalization of sacran, as shown in Figure 7B, calcium particles were formed due to electric field application to sacran aqueous solution. The phenomenon might be derived from calcium ions condensed by an electric field and deposited onto the film. On the other hand, these solvents did not observe such a calcium compound deposition phenomenon over ethylene glycol and 1,2-propanediol solutions, due to the lack of dissociation of calcium ion pairs with carboxylates and sulfonates.

**Figure 8:**
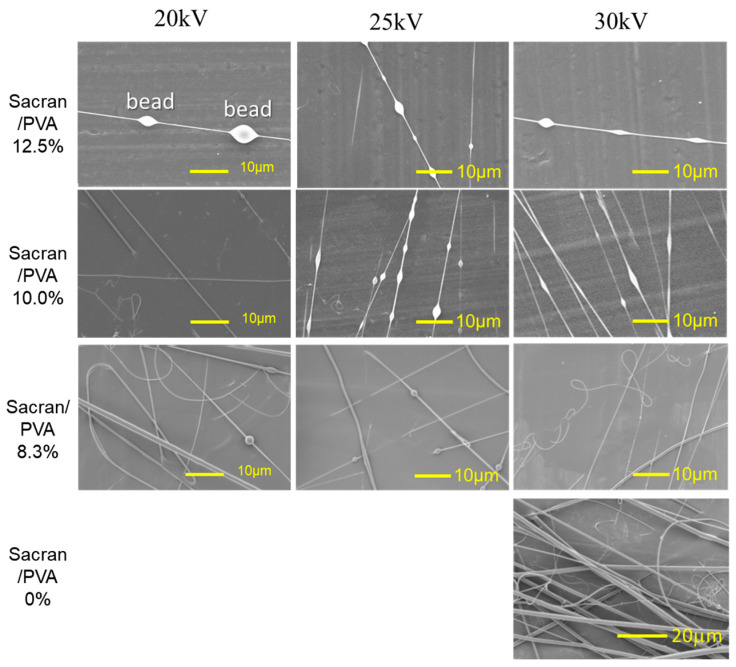
SEM images of polymer composites of sacran with PVA with various compositions spun in water. The top values are electric field values applied to sacran/PVA solutions. The left image is sacran composition to PVA. Beads appeared at high sacran composition and low electric field.

Overall, it was concluded that sacran microfibers should not be produced using a pure solvent. We used PVA to assist the sacran microfiber formation in aqueous solutions because PVA electrospun fibers can be easily obtained [41]. The electrospinning condition was optimized for PVA without sacran because the selected PVA has a high *MW* spinning grade in low concentrations, to match the concentration of highly viscous sacran solution (0.5%). As a result of optimization, the concentration of PVA had to be 2.0% or more and the applied voltage was 20 kV or more. Electrospinning of three mixtures with compositions of sacran, specifically, PVA = 0.25%: 2% (sacran percentage to PVA is 12.5%), 0.25%: 2.5% (10.0%), and 0.25%: 3% (8.3%), were successfully electro-spun at applied voltages of 20, 25, and 30 kV, respectively. The SEM image of electrospun fibers placed on aluminum foil is shown in Figure 8. The representative image of PVA (3%, without sacran) taken at 30 kV is shown in the bottom right, revealing the formation of microfibers with a radius of 1–2 μm. When 8.3% of sacran was mixed into the PVA solution, almost homogeneous microfibers with a radius of about 1 μm were obtained. When the amount of sacran was further increased to 10%, fibers with a radius less than 1 μm were obtained at 20 kV. In the case of 25 kV and 30 kV, the microfibers were shaped like a bead necklace. The beads were hemispherical, about 2–3 μm long and 1–2 μm wide, and were present at intervals of 10 to 20 μm. At 12.5% of sacran, spherical beads were more frequently observed with a length of about 2–7 μm and a width of about 1–5 μm. Overall, it was found that the beads became more stretched as the applied voltage increased and the sacran concentration decreased. As mentioned above, to obtain clear microfibers without beads, the upper limitation of sacran composition to PVA was about 8.3%. There is a possibility that the bead necklace itself may transport substances from bead to bead, but this paper did not conduct research to that extent.

**Figure 9:**
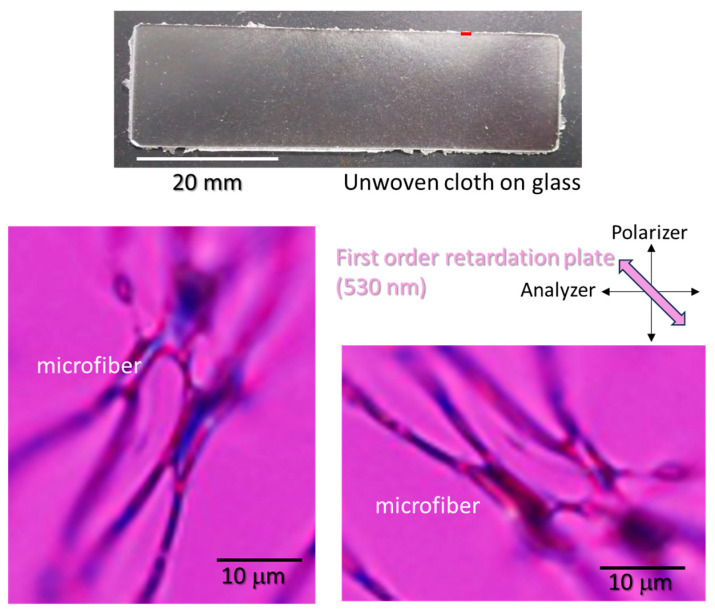
Non-woven cloth of polymer composite microfibers of sacran/PVA sample prepared in water (sacran/PVA 8.3 wt%, Voltage: 30 kV). The top picture is the appearance of microfibers on a glass plate. The bottom pictures are cross-polarizing microscopic images taken in the presence of a first-order retardation plate (530 nm) set at angle of 45 degree to the polarizer. The left image is presented at an angle of approximately 45 degrees from both the analyzer and polarizer, and the fibers placed from the bottom left to the top right are colored blue, while the sample color is orange when the fibers placed from the bottom right to the top left. The observation indicates that the fiber has positive birefringence.

The orientation of the microfibers obtained under conditions of 10% sacran and 30 kV was observed using a polarizing microscope. The sample was spun on a slide glass placed on aluminum foil (Figure 9). The appearance of the glass has become a little cloudy (top picture). A polarized light microscopy image of the part near the tip of this glass is shown below. This observation was taken in the presence of a first-order retardation plate (λ 530 nm) installed between the sample and the analyzer. The image on the left shows the microfibers present at an angle of approximately 45 degrees from both the analyzer and polarizer, and the fibers placed from the bottom left to the top right are colored blue. Also, this photo was taken after rotating this sample 90 degrees to the left, and it can be observed that the sample color changed to orange. The observation indicates that this sample has a positive birefringence. When the birefringence is positive, the sugar chains are oriented parallel to the fiber axis, and it is thought that they are oriented along the fiber axis together with PVA. It can be speculated that the orientation of sacran induced that of PVA microfibers. Since PVA is not easily oriented in an amorphous state, it is important that it could be oriented simply by introducing about 10% of sacran. Hence, adding just a small amount of sacran has a strong effect on the orientation of the matrix polymer chains. The resulting microfibers easily absorbed moisture, which can be regarded as responsiveness to humidity stimulus. In the future, we plan to evaluate stimuli-responsiveness such as humidity-responsive or photoresponsive materials.

In conclusion, the H-type cation-exchange resin for heterogeneous acid hydrolysis of sacran gives a short-time exposition of concentrated acid to sacran chains, which was extracted from ECM gels of *Aphanothece sacrum*. The oligomeric fraction with a well-defined structure was obtained from the supernatant. GC-MS and GC revealed that the oligomer had a main sugar ratio, Glc:Xyl:Rha, of 7 (=3 + 4):3:4, from which it was speculated that the sacran structure includes rhamnoglucan and xyloglucan, which is generally very rigid enough to exhibit LC. This structure must then be an origin for LC supercoiled assembly. The structure motivated us to prepare the sacran microfibers by electrospinning but sacran solutions in polyhydroxy alcohols gave only oriented films. It was noteworthy that the film formed by the electrospinning machine was still oriented, similar to a film cast over an aqueous solution by conventional drying. When poly(vinyl alcohol), PVA, was used to assist the microfiber formation, the polymer-composite microfibers of sacran with PVA were prepared in the molecularly oriented state. Since PVA and sacran are both cross-linked after fabrication by freeze–thaw and annealing, respectively, the orientated microfibers would be used as stimuli-responsive hydrogel materials in the future.

## 3. Materials and Methods

### 3.1. Material

Sacran is a macromolecular polysaccharide extracted from the extracellular matrix; it was obtained by following a previously described procedure [9]. The biomaterial samples were washed with pure water to remove hydrophilic impurities. Further, the water-washed samples were soaked in isopropanol overnight and then collected by filtration to remove more hydrophobic impurities than mentioned above. The isopropanol-washed samples were added to 0.1 M NaOH at around 100·°C and agitated until completely dissolved. The aqueous solution was neutralized to a pH of 6–7. Subsequently, the concentrated solution was filtered with cloth to remove undissolved impurities. Further, the viscous solution was slowly poured into pure isopropanol to precipitate white fibrils. The fibrils were collected and washed with isopropanol several times to eliminate impurities. The fibril sacran was further dried at 60 °C for several minutes.

Sodium alginate (viscosity: 800–900 mPa s (1% in water)) which was used for the parent polysaccharide of external standards, guluronic acid and mannuronic acid, was purchased from Tokyo Chemical Industry, Tokyo, Japan, and used as received. Concentrated hydrochloric acid (HCl) and trifluoroacetic acid (TFA) were purchased from Kanto Chemical Co., Ltd., Tokyo, Japan, and used after being diluted for methanolysis and acid hydrolysis as part of the GC-MS pretreatment. Methanol and acetyl chloride were purchased from Kanto Chemical Co., Ltd., Tokyo, Japan, and used as received. PVA (JP-50HH) with an average repeating unit number of 5000 (saponification degree > 98 mol%) was dedicated by Japan Vam & Poval Co., Ltd., Osaka, Japan was used as received. Monosaccharides and other sugar standards for GC-MS and GC were purchased from Tokyo Chemical Industry, Tokyo, Japan, and used as received. Cation exchange resin (DOWEX 50W-X8, 50–100 mesh, Merck KGaA, Darmstadt, Germany) was used after treatment by HCl (Tokyo Chemical Industry, Tokyo, Japan).

### 3.2. Methods

#### 3.2.1. Acid Hydrolyses and Fractionation

H-type cation exchange resins, which were pre-treated with 2N HCl, were placed into the sacran aqueous solution (1 g, 0.5 wt%). The mixture was mildly agitated to exchange sacran sodium ions into H^+^ to form a jelly matter of sacran. The jelly matter was collected by filtration and a fraction was isolated as a gel with the ion exchange resins (Fraction A) under vacuum. The filtrate was collected and condensed by evaporation, and the resulting viscose solution was poured into methanol. Fraction A was deposited as fibrils in methanol (Fraction B), and the part that was soluble in methanol was isolated (Fraction C).

#### 3.2.2. Monosaccharide Analyses

The methanolysis and trimethylsilylation treatments were created for the determination of the constituent monosaccharides by gas chromatography–mass spectrometry (GC-MS) and GC as follows. First, 1 N HCl-MeOH was prepared by mixing dry methanol (50 mL) with acetyl chloride (3.6 mL); this was added to an obtained fraction (1 mg) and heated to 70 °C and stirred for 16 h. The methanolized sample was evaporated and washed with t-BuOH three times. Then, the samples were trimethylsilylated with TMSI-C (200 uL) at ambient temperature for 1 h, for analyzing constituent monosaccharides by GC-MS. Fraction B was hydrolyzed using 4 M TFA at 110 °C for 4 h and then methanolized for 24 h based on an analogous procedureto a methanol-soluble fraction. Further, monosaccharides used as external standards such as Glc, Man, Gal, Rha, Fuc, Ara, Xyl, GlcN, GalN, GlcNAc, GalNAc, GalA, GlcA, Rib, Mur, and MurNAc were also methanolized and trimethylsilylate. For guluronic acid (GulA) and mannuronic acid (ManA), the alginate was hydrolyzed using 4M TFA at 110 °C for 48 h and methanolized for 44 h to reach the external standards of GulA and ManA.

The constituent monosaccharides were analyzed by GC (GC-18A, Simadzu, Kyoto, Japan) and GC-MS apparatus (Trace DSQ, Termo Fisher Scientific Inc., Waltharm, MA, USA) equipped with a 30 m × 0.25 mm i.d. fused-silica capillary column coated with 0.25-μm TC-1 (GL Sciences, Tokyo, Japan). The GC-MS flow speed of He gas was 1 mL/min, and the injector temperature was 200 °C. The column temperature program at onset was 80 °C, increasing to 260 °C at a rate of 6 °C/min. The transfer line and the ion source were both kept at a temperature of 250 °C, whereas the GC injector was kept at a temperature of 200 °C. The flame ionization detection was kept at a temperature of 250 °C. The column was kept at a temperature of 140 °C for 2 min, then raised by a rate of 8 °C/min to 260 °C, which was maintained for 13 min. The carrier N_2_ gas was kept at a pressure of 100 kPa. Moreover, the analyses of GC and GC-MS revealed the absence of GulA, ManA, or Rib as sugar monosaccharides of sacran, suggesting that sacran has a unique polysaccharide chain different from seaweed-derived chains, or that it is not contaminated with nucleic acids.

#### 3.2.3. Electrospinning

After dissolving 0.5 g of sacran into 100 mL of each solvent, the mixture was stirred at 60 °C for 48 h using a hot stirrer to prepare a 0.5 wt% sacran solution. Afterward, impurities in the solution were removed using a centrifuge (at 3000 rpm for 1 h at 20 °C, Avanti HP-26 XP (BECKMAN COULTER, Brea, CA, USA)). This operation was repeated three times in total to obtain a clear sacran solution. The prepared 0.5 wt% sacran solution was put into a syringe as a sample solution and installed in the electrospinning machine NANON (MECC, Tosu, Japan). Regarding electrospinning conditions, we focused on voltage and investigated the optimal conditions for forming fibers. Three types of voltage were used, specifically 20 kV, 25 kV, and 30 kV, and three different inner needle diameters (22 G (0.4 mm)) attached to the syringe were used. In addition, electrospinning was performed with the sample extrusion speed and the distance from the tip of the needle to the collector where the fibers are collected (collector distance) fixed at 1.0 mL/h and the collector distance at 10 cm. After electrospinning, the sample on the collector was vacuum-dried for 24 h. Sacran aqueous solution with a concentration of 0.5 wt% was used as a sample; for PVA-assisted spinning, PVA aqueous solution with a concentration range of 3–5 wt% was mixed with sacran solution to use as samples.

#### 3.2.4. Polarized Microscopy

Cross-polarizing microscopy was used to observe the orientation of microfibers using a microscope (BX51, Olympus, Tokyo, Japan) equipped with a CCD camera (DP80, Olympus, Tokyo, Japan). A first-order retardation plate (530 nm) was inserted into the light path to identify the orientation direction.

#### 3.2.5. Scanning Electron Microscopy

A scanning electron microscope (SEM, JEOL, JCM-6000PLUS, Tokyo, Japan) was used to investigate the sacran film and microfibers formed in the electrospinning machine. The samples were mounted onto metal stubs using carbon tape. The stubs were then coated with Pt-Pd using a sputtering machine. Energy-dispersive X-ray spectroscopy (EDX) is carried out for elemental analysis.

## Figures and Tables

**Table 1:** Solubility tests of sacran ^1^.

Water	MeOH	EtOH	Acetone	Ethylene Glycol	1,2-Propanediol	Glycerol	DMSO
+	−	−	−	+	+	+	+

^1^ + soluble, − insoluble.

## Data Availability

The data presented in this study are openly available in article.

## References

[1] Stortz C.A., Cerezo A.S. (2000). Novel findings in carrageenans, agaroids and “hybrid” red seaweed galactans. Curr. Top. Phytochem..

[2] Khanra S., Mondal M., Halder G., Tiwari O.N., Gayen K., Bhowmick T.K. (2018). Downstream processing of microalgae for pigments, protein and carbohydrate in industrial application: A review. Food Bioprod. Process..

[3] Lee K.Y., Mooney D.J. (2012). Alginate: Properties and biomedical applications. Prog. Polym. Sci..

[4] Li B., Lu F., Wei X., Zhao R. (2008). Fucoidan: Structure and Bioactivity. Molecules.

[5] Hori K., Ueno T.-M., Okita T. (1992). Absorption of color additives and settling volume in water of blue-green alga, ishikurage (Nostoc commune). Plant Foods Hum. Nutr..

[6] Kabata K., Okamoto C., Sasada N., Ono M., Igoshi K., Kobayashi H., Masuoka C., Ito Y. (2005). 日本固有種ラン藻・スイゼンジノリ(Aphanothece sacrum (Sur.) Okada)の培養および構成単糖と機能性の検索. Kyushu Tokai Daigaku Nogakubu Kiyo.

[7] Watanabe F., Miyamoto E., Fujita T., Tanioka Y., Nakano Y. (2006). Characterization of a Corrinoid Compound in the Edible (Blue-Green) Alga, Suizenji-nori. Biosci. Biotechnol. Biochem..

[8] Ogura F., Hayashi K., Lee J.B., Kanekiya K., Hayashi T. (2010). Evaluation of an Edible Blue-Green Alga, *Aphanothece sacrum*, for Its Inhibitory Effect on Replication of Herpes Simplex Virus Type 2 and Influenza Virus Type A. Biosci. Biotechnol. Biochem..

[9] Fujishiro T., Ogawa T., Matsuoka M., Nagahama K., Takeshima Y., Hagiwara H. (2004). Establishment of a Pure Culture of the Hitherto Uncultured Unicellular Cyanobacterium Aphanothece sacrum, and Phylogenetic Position of the Organism. Appl. Environ. Microbiol..

[10] Oku N., Hana S., Matsumoto M., Yonejima K., Tansei K., Isogai Y., Igarashi Y. (2017). Two new sacrolide-class oxylipins from the edible cyanobacterium *Aphanothece sacrum*. J. Antibiot..

[11] Uchida Y., Maoka T., Palaga T., Honda M., Tode C., Shimizu M., Waditee-Sirisattha R., Kageyama H. (2023). Identification of Desiccation Stress-Inducible Antioxidative and Antiglycative Ultraviolet-Absorbing Oxylipins, Saclipin A and Saclipin B, in an Edible Cyanobacterium *Aphanothece sacrum*. J. Agric. Food Chem..

[12] Ren S., Gao Y., Wang L., Qiu C., Yang L., Li L., Xiao Y., Xiao N., Liao L., Zuo Z. (2022). Sacran polysaccharide improves atopic dermatitis through inhibiting Th2 type immune response. Life Sci..

[13] Budpud K., Okeyoshi K., Kobayashi S., Okajima M.K., Kaneko T. (2022). Super-Moisturizing Materials from Morphological Deformation of Suprapolysaccharides. Macromol. Rapid Commun..

[14] Fraser J.R.E., Laurent T.C., Laurent U.B.G. (1997). Hyaluronan: Its nature, distribution, functions and turnover. J. Intern. Med..

[15] Kim J.-H., Yoo S.-J., Oh D.-K., Kweon Y.-G., Park D.-W., Lee C.-H., Gil G.-H. (1996). Selection of a Streptococcus equimutant and optimization of culture conditions for the production of high molecular weight hyaluronic acid. Enzym. Microb. Technol..

[16] Vincent J.F.V. (1999). From cellulose to cell. J. Exp. Biol..

[17] Giraud-Guille M.M. (1988). Twisted plywood architecture of collagen fibrils in human compact bone osteons. Calcif. Tissue Int..

[18] Badry R., El-Nahass M.M., Nada N., Elhaes H., Ibrahim M.A. (2023). Structural and UV-blocking properties of carboxymethyl cellulose sodium/CuO nanocomposite films. Sci. Rep..

[19] Sundaresan N., Suresh C.H., Thomas T., Thomas T.J., Pillai C.K.S. (2008). Liquid crystalline phase behavior of high molecular weight DNA: A comparative study of the influence of metal ions of different size, charge and binding mode. Biomacromolecules.

[20] Budpud K., Okeyoshi K., Okajima M., Kaneko T. (2020). Vapor-sensitive materials from polysaccharide fibers with self-assembling twisted microstructures. Small.

[21] Ali M.A., Singh M., Zhang S., Kaneko D., Okajima M.K., Kaneko T. (2024). Metal-Assisted Injection Spinning of Ultra Strong Fibers from Megamolecular LC Polysaccharides. Polymers.

[22] Yanaki T., Norisuye T., Teramoto A. (1984). Cholesteric Mesophase in Aqueous Solutions of a Triple Helical Polysaccharide Scleroglucan. Polym. J..

[23] Dong X.M., Gray D.G. (1997). Effect of Counterions on Ordered Phase Formation in Suspensions of Charged Rodlike Cellulose Crystallites. Langmuir.

[24] Oertel R., Kulicke W.M. (1991). Viscoelastic properties of liquid-crystals of aqueous biopolymer solutions. Rheol. Acta.

[25] Flory P.J., Platé N.A. (1984). Molecular theory of liquid crystals. Liquid Crystal Polymers I. Advances in Polymer Science.

[26] De Gennes P.G. (1995). Physics of Liquid Crystals.

[27] Ngatu N.R., Okajima M.K., Yokogawa M., Ryoji Hirota R., Eitoku M., Muzembo A.B., Dumavibhat N., Takaishi M., Sano S., Kaneko T. (2012). Anti-inflammatory effects of sacran, a novel polysaccharide from Aphanothece sacrum, on 2,4,6-trinitrochlorobenzene–induced allergic dermatitis in vivo. Ann. Allergy Asthma Immunol..

[28] Doi M., Sagawa Y., Tanaka T., Mizutani T., Okano Y., Masaki H. (2018). Defensive Effects of a Unique Polysaccharide, Sacran, to Protect Keratinocytes against Extracellular Stimuli and Its Possible Mechanism of Action. Biol. Pharm. Bull..

[29] Doi M., Sagawa Y., Sasano K., Tanaka T., Mizutani T., Okano Y., Masaki H. (2019). Protective Effects of Sacran, a Natural Polysaccharide, Against Adverse Effects on the Skin Induced by Tobacco Smoke. J. Cosmet. Sci..

[30] Motoyama K., Tanida Y., Sakai A., Higashi T., Kaneko S., Arima H. (2018). Anti-allergic effects of novel sulfated polysaccharide sacran on mouse model of 2,4-Dinitro-1-fluorobenzene-induced atopic dermatitis. Int. J. Biol. Macromol..

[31] Puluhulawa L.E., Joni I.M., Mohammed A.F.A., Arima H., Wathoni N. (2021). The Use of Megamolecular Polysaccharide Sacran in Food and Biomedical Applications. Molecules.

[32] Matsuda S., Sugawa H., Shirakawa J., Ohno R., Kinoshita S., Ichimaru K., Arakawa S., Nagai M., Kabata K., Nagai R. (2017). *Aphanothece sacrum* (Sur.) Okada Prevents Cataractogenesis in Type 1 Diabetic Mice. J. Nutr. Sci. Vitaminol..

[33] Wada H., Gombos Z., Murata N. (1990). Enhancement of chilling tolerance of a cyanobacterium by genetic manipulation of fatty acid desaturation. Nature.

[34] Guo H., Yi W., Song J.K., Wang P.G. (2008). Current understanding on biosynthesis of microbial polysaccharides. Curr. Top. Med. Chem..

[35] Cornelis P. (2008). Pseudomonas: Genomics and Molecular Biology.

[36] Tamaru Y., Takani Y., Yoshida T., Sakamoto T. (2005). Crucial Role of Extracellular Polysaccharides in Desiccation and Freezing Tolerance in the Terrestrial Cyanobacterium Nostoc commune. Appl. Environ. Microbiol..

[37] Hill D.R., Keenan T.W., Helm R.F., Potts M., Crowe L.M., Crowe J.H. (1997). Extracellular polysaccharide of Nostoc commune (Cyanobacteria) inhibits fusion of membrane vesicles during desiccation. J. Appl. Phycol..

[38] Okajima M.K., Sornkamnerd S., Kaneko T. (2018). Development of Functional Bionanocomposites using Cyanobacterial Polysaccharides. Chem. Rec..

[39] Julian J.D., Zabotina O.A. (2022). Xyloglucan Biosynthesis: From Genes to Proteins and Their Functions. Front. Plant Sci..

[40] Dhara S., Chenchula S.R., Chakraborty K., Valluru L., Surabhi G. (2024). Sulfated rhamnoglucan heteropolysaccharide of Spirulina platensis attenuates methimazole-induced hypothyroidism in rats. Algal Res..

[41] Yano T., Higaki Y., Tao D., Murakami D., Kobayashi M., Ohta N., Koike J., Horigome M., Masunaga H., Ogawa H. (2012). Orientation of poly(vinyl alcohol) nanofiber and crystallites in non-woven electrospun nanofiber mats under uniaxial stretching. Polymer.

